# The *Arabidopsis pop2-1 *mutant reveals the involvement of GABA transaminase in salt stress tolerance

**DOI:** 10.1186/1471-2229-10-20

**Published:** 2010-02-01

**Authors:** Hugues Renault, Valérie Roussel, Abdelhak El Amrani, Matthieu Arzel, David Renault, Alain Bouchereau, Carole Deleu

**Affiliations:** 1 INRA - Agrocampus Ouest - Université de Rennes 1, UMR 118 Amélioration des Plantes et Biotechnologies Végétales, F-35653, Le Rheu cedex, France; 2 CNRS - Université de Rennes 1, UMR 6553 EcoBio, Campus de Beaulieu, F-35042 Rennes cedex, France; 3 UMR 7208 BOREA, Station de Biologie Marine, Muséum National d'Histoire Naturelle, Place de la Croix, F-29900 Concarneau, France

## Abstract

**Background:**

GABA (γ-aminobutyric acid) is a non protein amino acid that has been reported to accumulate in a number of plant species when subjected to high salinity and many other environmental constraints. However, no experimental data are to date available on the molecular function of GABA and the involvement of its metabolism in salt stress tolerance in higher plants. Here, we investigated the regulation of GABA metabolism in *Arabidopsis thaliana *at the metabolite, enzymatic activity and gene transcription levels upon NaCl stress.

**Results:**

We identified the GABA transaminase (GABA-T), the first step of GABA catabolism, as the most responsive to NaCl. We further performed a functional analysis of the corresponding gene *POP2 *and demonstrated that the previously isolated loss-of-function *pop2-1 *mutant was oversensitive to ionic stress but not to osmotic stress suggesting a specific role in salt tolerance. NaCl oversensitivity was not associated with overaccumulation of Na^+ ^and Cl^- ^but mutant showed a slight decrease in K^+^. To bring insights into *POP2 *function, a promoter-reporter gene strategy was used and showed that *POP2 *was mainly expressed in roots under control conditions and was induced in primary root apex and aerial parts of plants in response to NaCl. Additionally, GC-MS- and UPLC-based metabolite profiling revealed major changes in roots of *pop2-1 *mutant upon NaCl stress including accumulation of amino acids and decrease in carbohydrates content.

**Conclusions:**

GABA metabolism was overall up-regulated in response to NaCl in *Arabidopsis*. Particularly, GABA-T was found to play a pivotal function and impairment of this step was responsible for a decrease in salt tolerance indicating that GABA catabolism was a determinant of *Arabidopsis *salt tolerance. GABA-T would act in salt responses in linking N and C metabolisms in roots.

## Background

Salt stress affects crop productivity worldwide, especially in irrigated lands [1], and can thus lead to dramatic consequences in food availability. Hence, determinants of plant salt tolerance are intensively investigated to identify targets for plant breeding and to create salt tolerant varieties. Three cellular components of salt tolerance have been proposed in plants: (*i*) osmotic stress tolerance, (*ii*) Na^+ ^exclusion capacity and (*iii*) tissue tolerance to Na^+ ^accumulation [2]. Unlike halophytic species, the glycophytic plant-model *Arabidopsis thaliana *is sensitive to moderate levels of NaCl. This has raised the question of its relevance in salt tolerance studies [3]. However, thanks to genetic and molecular tools developed around this species, several genes involved in plant salt tolerance have been highlighted. Thus, many mutants or transgenic lines of *A*. *thaliana *were shown to display differential levels of NaCl tolerance and this mostly concerned genes involved in ion transport [4-8], detoxication processes [9,10] or metabolite biosynthesis [11,12].

Among stress-responsive metabolites, γ-aminobutyric acid is of special interest since the molecule accumulates in response to a wide range of environmental stimuli [13] although its function in plants is still a matter of debate [14,15]. GABA is a widespread non protein amino acid, from prokaryotes to eukaryotes. It has been first discovered in plants in the middle of the 20^th ^century [16] but rapidly attention shifted to its signaling function in mammals central nervous system as a neurotransmitter. In plants, speculative functions have been attributed to GABA metabolism such as osmoregulation [17] and glutamate homeostasis control [18]. Moreover, it has been demonstrated to participate to pH regulation [19,20] and bypass of TCA cycle [21]. GABA has also been shown to act as a signaling molecule in plants as reported for nitrate uptake modulation [22], *14-3-3 *genes regulation [23] and pollen tube growth and guidance [24].

**Figure 1:**
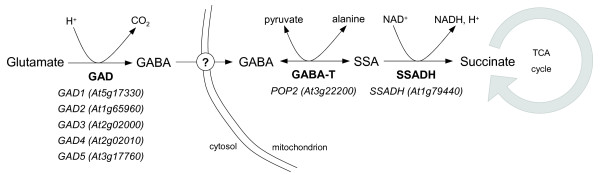
**Schematic representation of the GABA metabolic pathway in *Arabidopsis thaliana***. GAD, glutamate decarboxylase; GABA-T, GABA transaminase; SSA, succinic semialdehyde; SSADH, succinic semialdehyde dehydrogenase. For each enzyme, the corresponding genes loci are shown.

In plants and animals, GABA metabolism is sum up in a three-enzyme-pathway that takes place in two cellular compartments (figure 1). GABA is mainly synthesized from L-glutamate owing to the activity of the cytosolic glutamate decarboxylase (GAD, EC 4.1.1.15). GABA is then transported into the mitochondrion to be catabolized by the GABA transaminase (GABA-T, EC 2.6.1.19) which converts GABA to succinic semialdehyde (SSA) [25]. Subsequently, SSA is oxidized by the mitochondrial succinic semialdehyde dehydrogenase (SSADH, EC 1.2.1.16) to produce succinate [26]. Alternatively, SSA can also be reduced in the cytosol *via *the activity of the γ-hydroxybutyrate dehydrogenase (GHBDH, EC 1.1.1.61) that produces γ-hydroxybutyrate (GHB) [27].

Most of attention has been focused on GABA synthesis under environmental stress owing to changes of catalytic properties of plants GAD depending on cytosolic pH and activity of Ca^2+^/calmodulin complex [28,29], two known stress-modulated factors [17]. On this basis, it has been hypothesized that GABA level could be mainly controlled by the rate of its synthesis. However, isolation and characterization of *Arabidopsis *GABA-T deficient mutants demonstrated that GABA levels could also result from the rate of its degradation [24,30,31]. *Arabidopsis *genome contains only one GABA-T encoding gene (*At3 g22200*; figure 1) [25], subsequently termed *POP2 *(*Pollen-Pistil Incompatibility 2*) [24], whereas 5 genes putatively encode GAD (*GAD1-5*; figure 1) [32]. POP2 uses pyruvate as GABA amino group acceptor (GABA-TP activity) [25], while in mammals GABA-T exclusively uses 2-ketoglutarate as amino group acceptor (GABA-TK activity) [33]. Recently, it has been shown that POP2 can also uses glyoxylate as amino acceptor and thus produces glycine [34]. *POP2 *gene product is a 55.2 kDa polypeptide with a pyridoxal-5-phosphate binding domain and a mitochondrial peptide signal [34], and shares little homology with non-plant *GABA-T *genes [25]. In *A.thaliana*, *POP2 *gene was linked to responsiveness to volatile *E*-2-hexenal [30], alanine accumulation occurring in roots during hypoxia [35] and growth and guidance of pollen tubes [24].

In this study, we investigated the regulation of GABA metabolism upon NaCl treatments in *A. thaliana *at the metabolite, enzymatic activity and gene transcription levels. We identified the GABA-T step as a key point of regulation of GABA metabolism and further performed a functional analysis of the *POP2 *gene that encodes GABA-T.

## Results

### GABA-T is the most responsive step of GABA metabolism upon NaCl stress in *A. thaliana*

**Figure 2:**
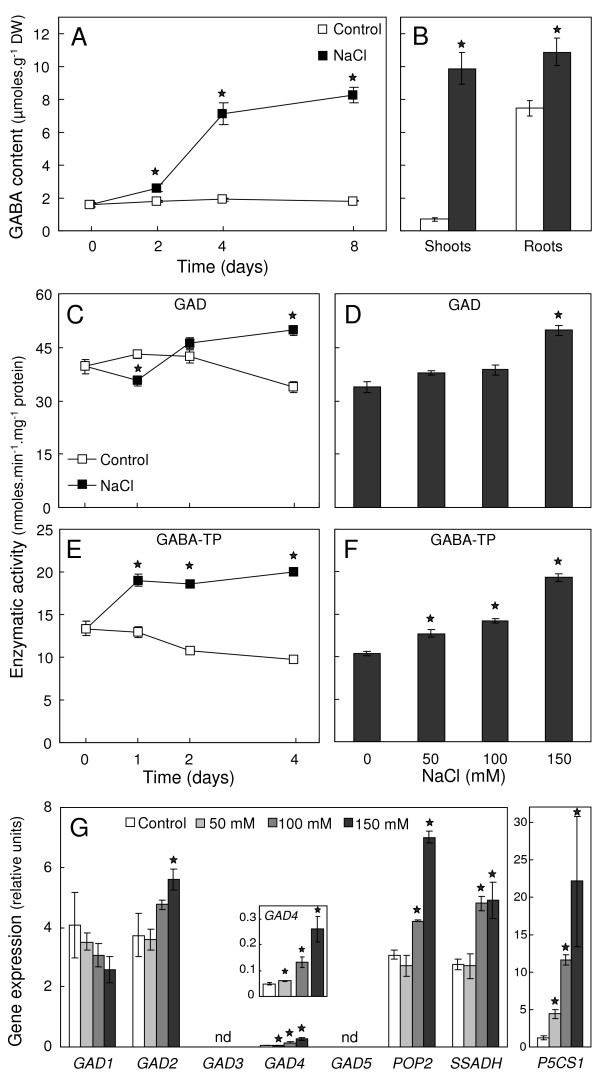
**GABA metabolism regulation upon NaCl treatment**. Ten-day-old plantlets of wild-type (WT, L*er *accession) grown on agar medium were transferred to agar medium supplemented, or not (Control), with NaCl. **(A-B) **Time-course and organ partitioning of GABA content during NaCl treatment. GABA content was determined either in whole plantlets treated with 150 mM NaCl over an 8-day-period (A) or in shoots and roots of plantlets after 4 days of treatment with 150 mM NaCl (B). Results are the mean ± S.E. of 3 independent replicates. **(C-F) **Time-course and dose-response of GAD and GABA-TP activities upon NaCl. Glutamate decarboxylase activity (GAD, D-E) and GABA transaminase activity using pyruvate as GABA amino group acceptor (GABA-TP, F-G) were determined in entire plantlets either over a 4-day-period of treatment with 150 mM NaCl (D and F) or after 4 days of treatment with increasing concentration of NaCl (E and G). Results are the mean ± S.E. of 4-10 independent replicates. **(G) **Dose-response of GABA metabolism genes to increasing concentration of NaCl after 24 h of treatment. Total RNA was isolated from whole plantlets and served to gene expression analysis of the five glutamate decarboxylase (*GAD1-5*), the GABA transaminase (*POP2*), the succinate semialdehyde dehydrogenase (*SSADH*) and the well-known stress-induced Δ^1^-pyrroline-5-carboxylate synthetase 1 (*P5CS1*). Results are the mean ± S.E. of 3 independent replicates. nd, not detected. Stars indicate a significant difference with control according to non-parametric Mann-Whitney *U*-test (*P *< 0.05)

No data specifically devoted to description of GABA level changes under NaCl stress conditions are to date available in *A. thaliana*. Hence, we followed the kinetics of GABA level changes and its organ partitioning in wild-type plantlets (WT) subjected to 150 mM NaCl treatment. Figure 2A shows that GABA readily accumulated during NaCl treatment in *A. thaliana *at the whole-plant level. After 4 days of treatment, GABA content reached 3.8-fold higher level in NaCl-treated plantlets than in control ones (7.1 vs 1.9 μmoles.g^-1 ^DW; figure 2A). Under control conditions, GABA was shown to be much more abundant in root tissues than in shoot tissues (7.5 vs 0.7 μmoles.g^-1 ^DW; figure 2B) whereas, after 4 days of treatment with NaCl, shoot and root tissues exhibited about equal amount of GABA (9.9 vs 10.9 μmoles.g^-1 ^DW). Shoots of NaCl-treated plantlets were actually shown to accumulate 14-fold more GABA than control ones while roots accumulated only 1.5-fold more GABA (figure 2B).

GAD and GABA-TP catalytic activities were determined *in vitro *in WT plantlets subjected to NaCl treatments to decipher biochemical determinants of GABA accumulation. GAD activity showed surprising variations (figure 2C) in response to NaCl treatment. It was thus found to be significantly decreased in plantlets treated for 24 h with 150 mM NaCl while, after 4 days of treatment, it reached 1.5-fold higher level than in control plantlets (49.7 vs 33.9 nmoles.min^-1^.mg^-1 ^protein; figure 2C). GAD activity was not shown to be significantly different in plantlets treated for 4 days with 50 mM and 100 mM NaCl (figure 2D). Figure 2E shows that GABA-TP activity increased rapidly in response to treatment with 150 mM NaCl. In plantlets treated for 4 days, GABA-TP activity was 2.1-fold higher than in control plantlets (20.0 vs 9.7 nmoles.min^-1^.mg^-1 ^protein; figure 2E) and was actually found to respond to NaCl in a dose-dependent manner (figure 2F).

It was of interest to ascertain whether enzymes activities were correlated with changes in transcriptional activity of GABA metabolism genes. To achieve this objective, genes expression analysis was performed by qRT-PCR on total RNA isolated from entire WT plantlets treated for 24 h with increasing concentrations of NaCl. Primers were designed in order to ensure specific amplification (see Methods section and Additional file 1). As shown in figure 2G, only the expression of 3 *GAD *genes was detectable under our experimental conditions. *GAD1 *and *GAD2*, the two most expressed paralogs, showed contrasted expression changes in response to NaCl treatments. *GAD1 *expression, which is root-specific [36], was shown to be gradually restricted as far as NaCl concentration increased. On the opposite, *GAD2 *expression, which is present in all parts of plant [37], was significantly enhanced when the salt level exceeded 100 mM (figure 2G). *GAD4 *expression was much lower than those of the two other GAD isoforms but it was found to be significantly enhanced in NaCl-treated plantlets (figure 2G). *GAD4 *expression was indeed 5.3-fold higher in plantlets treated for 24 h with 150 mM NaCl than in control plantlets. In such plantlets, *POP2 *expression was 2.3-fold higher than in control plantlets (figure 2G) and was actually found to be the most expressed gene of the GABA metabolism suggesting a pivotal function in salt stress responses. Interestingly, *SSADH *expression was also enhanced at 100 mM and 150 mM NaCl concentrations (figure 2G) indicating that whole GABA catabolism was transcriptionally up-regulated upon NaCl treatment. In parallel, expression of Δ^1^-*pyrroline-5-carboxylate synthetase 1 *(*P5CS1*), a well-known salt stress-induced gene involved in proline synthesis [38], was shown to be gradually induced, thus validating our experimental conditions (figure 2G).

### The GABA-T deficient mutant *pop2-1 *is ovsersensitive to NaCl

**Figure 3:**
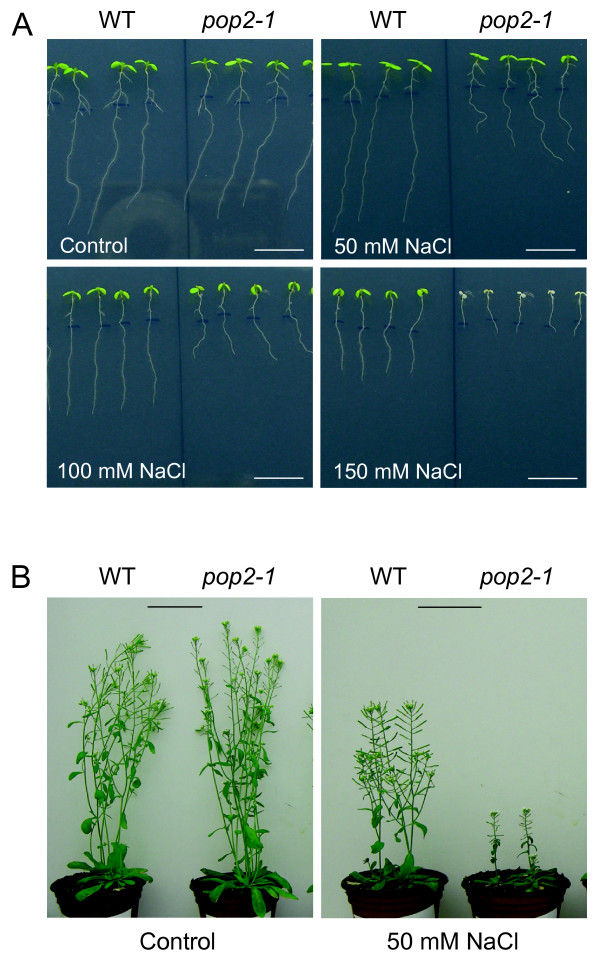
**Oversensitive phenotype of *pop2-1 *mutant in response to NaCl**. **(A) **Phenotype of 10-day-old plants treated for 6 days with, or without (control), 50, 100 and 150 mM NaCl. Scale bar = 1 cm. **(B) **Phenotype of 60-day-old plants grown on soil and alimented since their 14-day-old stage with the nutrient solution enriched, or not (control), with 50 mM NaCl. Scale bar = 5 cm.

**Figure 4:**
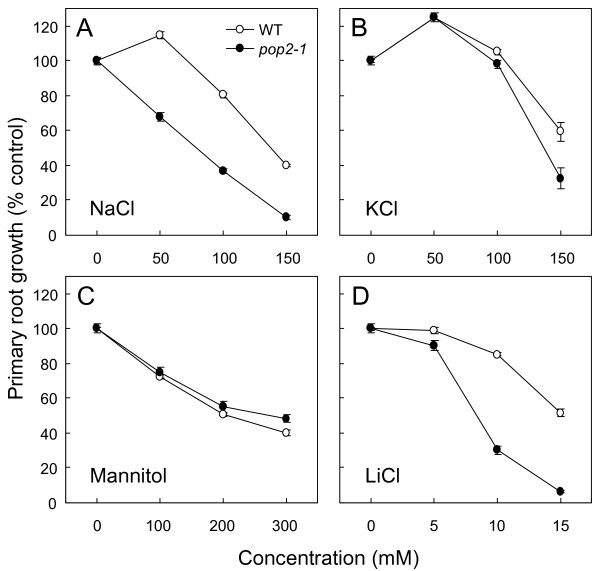
**Oversensitivity of *pop2-1 *mutant to ionic stress**. Four-day-old seedlings of WT and *pop2-1 *were transferred to agar medium supplemented with various concentrations of salts or osmoticum. After transfer, root apex was marked and primary root growth was recorded after 6 days. Primary root growth on agar medium supplemented with NaCl **(A)**, KCl **(B)**, Mannitol **(C) **and LiCl **(D)**. Results are the mean ± S.E. of measurements made on at least 16 plants distributed over three plates.

We tested the sensitivity to NaCl of the previously isolated GABA-T deficient *pop2-1 *mutant [24] on agar medium and under more physiological conditions in soil. In both case, NaCl treatment induced severe phenotype in the mutant, even death on agar medium supplemented with 150 mM NaCl, whereas no obvious difference occurred under control conditions between the mutant and its WT (figures 3A and 3B). NaCl sensitivity was more obvious at the root level since no clear symptoms appeared in aerial part of plants for NaCl concentrations below 150 mM (figure 3A). As a convenient way to decipher *pop2-1 *oversensitivity to NaCl, we compared primary root growths of *pop2-1 *mutant and WT on agar media supplemented with various salts or osmoticum. As shown in figure 4A, *pop2-1 *root growth was found to be oversensitive to NaCl. Unlike to WT, mutant root growth was indeed sharply reduced at 50 mM NaCl and decreased linearly as NaCl concentration increased in the medium (figure 4A). NaCl concentration that induced 50% inhibition of root growth (*I*_50_) was close to 81 mM for *pop2-1 *and 138 mM for WT. Furthermore, this response was mainly due to Na^+ ^because treatments with increasing concentration of KCl were less inhibitory for root growth of the mutant (*I*_50 _= 137 mM; figure 4B). The possibility of a pleiotropic sensitivity to toxic cations of *pop2-1 *was ruled out since the mutant did not display special phenotype in response to 1 mM spermidine and 100 μg/ml kanamycin (Additional file 2). In this context, it was of interest to verify whether *pop2-1 *root growth was also affected by osmotic stress. For this purpose, we used osmotically active concentrations of mannitol and osmotically non-active concentrations of the highly toxic LiCl. Thus, *pop2-1 *mutant did not appear to be oversensitive to mannitol (figure 4C) while LiCl induced a strong inhibition of *pop2-1 *root growth (*I*_50 _= 8.4 mM vs 15.2 mM for WT; figure 4D). These observations indicate that *pop2-1 *mutant is oversensitive to ionic stress, but not to osmotic stress.

**Figure 5:**
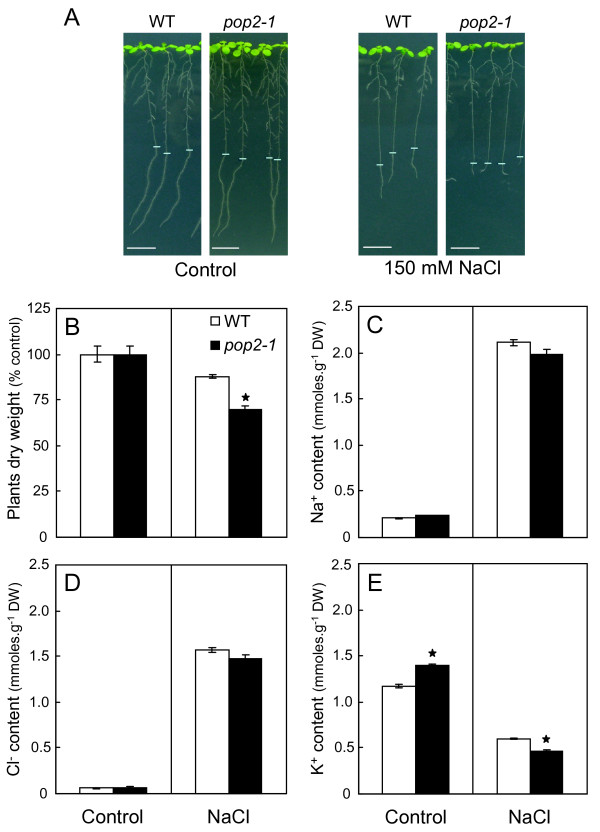
**Phenotypic and physiological characterization of *pop2-1 *upon NaCl treatment**. Ten-day-old plantlets of WT and *pop2-1 *mutant grown on agar medium were transferred for 4 days on agar medium supplemented, or not (Control), with 150 mM NaCl. For each condition, 15 entire plants were harvested for subsequent analysis. **(A) **Phenotype of plants at the end of NaCl treatment. Blue traits indicate primary root apex location at the onset of treatment. Scale bar = 1 cm. **(B) **Plants dry weight after NaCl treatment. Cl^- ^**(C)**, Na^+ ^**(D) **and K^+ ^**(E) **content of plantlets after NaCl treatment. Results are the mean ± S.E of 4 independent replicates. Stars indicate a significant difference with WT in the same condition according to non-parametric Mann-Whitney *U*-test (*P *< 0.05).

Treatment of 10-day-old plantlets with 150 mM NaCl for 4 days induced a greater growth inhibition in *pop2-1 *than in WT (30% vs 13% of growth inhibition respectively; figures 5A and 5B). The *pop2-1 *growth restriction was not associated with overaccumulation of Na^+ ^(figure 5C) or Cl^- ^(figure 5D) in plant tissues that might lead to a higher internal ionic stress. However, K^+ ^content was found to be significantly different between WT and *pop2-1 *mutant under both conditions (figure 5E). Thus, whereas K^+ ^content was significantly greater in *pop2-1 *than in WT under control conditions (1.4 vs 1.2 mmoles.g^-1 ^DW), *pop2-1 *exhibited a lesser K^+ ^content after NaCl treatment (0.46 vs 0.59 mmoles.g^-1 ^DW; figure 5E). Nevertheless, the K^+^/Na^+ ^ratio of *pop2-1 *mutant after NaCl treatment was not found to be significantly different from that of WT (0.24 ± 0.009 and 0.28 ± 0.007 respectively, *P *> 0.05, Mann-Whitney *U*-test; data not shown). To ascertain that the mutant was not impaired in K^+ ^uptake and transport, we germinated WT and *pop2-1 *seedlings on agar nutrient medium with low K^+ ^content (5 μM, 50 and 500 μM) and noted that *pop2-1 *grew as well as did the WT under low K^+ ^conditions (Additional file 3). Furthermore, the attempt to rescue *pop2-1 *phenotype on 150 mM NaCl medium by adding 20 mM KCl was unsuccessful (data not shown).

### Metabolic profiling of *pop2-1 *mutant reveals major changes in roots upon NaCl treatment

**Figure 6:**
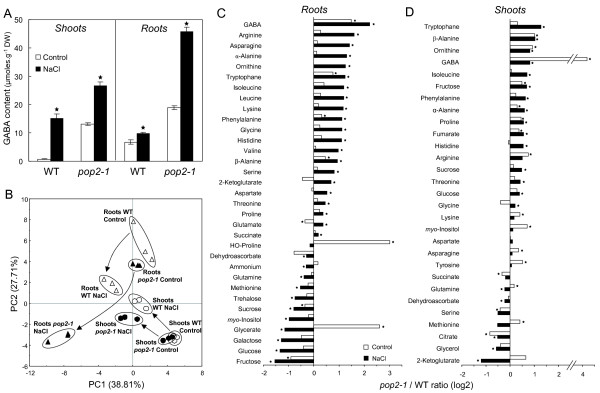
**Metabolic profiles of *pop2-1 *upon NaCl treatment**. Main polar metabolites occurring in roots and shoots of WT and *pop2-1 *were determined in 14-day-old plantlets treated for 4 days with 150 mM NaCl. Amino acids, excepted serine, were determined using Acquity UPLC system, other metabolites were determined using GC-MS system. **(A) **GABA content in *pop2-1 *mutant upon NaCl. **(B) **Principal component analysis of metabolite profiling data. Samples plot on the first two principal components (PCs) is shown. **(C-D) **Comparison of metabolite levels in WT and *pop2-1 *roots (C) and shoots (D). Only metabolites showing a significantly different content between *pop2-1 *and WT (Mann-Whitney *U*-Test, *P *< 0.05) in at least one condition (Control or NaCl) were considered. Quotients of mean content of *pop2-1 *(n = 3) over WT (n = 3) were plotted on a logarithmic scale (log2). Values < 0 represent a lower content in *pop2-1 *compared to WT; values > 0 represent a greater content in *pop2-1 *compared to WT. Stars indicate a significant difference between *pop2-1 *mutant and WT according to non-parametric Mann-Whitney *U*-test (*P *< 0.05).

Metabolic disorders that might be induced by GABA-T activity impairment were investigated by profiling the major primary polar metabolites occurring in shoots and roots of WT and *pop2-1 *after 4 days of treatment with 150 mM NaCl. A targeted analysis of GABA content in *pop2-1 *mutant and its WT was first performed and showed that mutant constitutively overaccumulated GABA under control conditions compared with WT, about 18-fold more in shoots and 2.8-fold more in roots (figure 6A). Under NaCl conditions, GABA reached high levels in *pop2-1 *mutant, especially in roots where the GABA content was close to 46 μmoles.g^-1 ^DW (figure 6A). Principal component analysis was then performed in order to extract meaningful information from the whole dataset. Thus, we were able to separate all conditions on the two first components (figure 6B), which were found to explain more than 66% of the dataset variability. WT and *pop2-1 *shoots metabolic profiles were shown to be very close under control conditions and also, to a lesser extent, under NaCl conditions (figure 6B). In contrast, metabolic profile of *pop2-1 *roots was clearly different from that of WT, especially after NaCl treatment as illustrated by the distance separating "Roots *pop2-1 *NaCl" cluster and "Roots WT NaCl" cluster (figure 6B). Among the 41 metabolites determined, 31 were shown to be present in a significantly different amount in *pop2-1 *roots after NaCl treatment (figure 6C). Interestingly, most of those that were more abundant in the mutant after NaCl treatment were amino acids while metabolites that were less abundant in the mutant were mostly carbohydrates (fructose, glucose, galactose, sucrose and trehalose; figure 6C). Surprisingly, succinate was shown to be significantly more abundant in roots of *pop2-1 *after NaCl treatment (figure 6C) although this compound could partly result from GABA degradation (figure 1). Other TCA cycle intermediates (citrate, fumarate, malate), except 2-ketoglutarate which was more abundant in *pop2-1 *after NaCl treatment (figure 6C), were not found to be present in a significantly different amount in roots of *pop2-1 *and WT (absolute values in Additional file 4) suggesting that TCA cycle activity was not fundamentally compromised upon NaCl stress in mutant roots. In shoots, metabolic disorders induced by NaCl treatment seemed to be less severe since metabolite ratio between *pop2-1 *and WT were not so far different than under control conditions except for tryptophan and 2-ketoglutarate (Figure 6D). Unlike roots, shoots of *pop2-1 *mutant were shown to accumulate more fructose, sucrose and glucose after NaCl treatment. Surprisingly, GABA did not belong to the most discriminant metabolites between WT and *pop2-1 *(cos^2 ^< 0.75; data not shown).

### *POP2 *expression pattern is reconfigured upon NaCl treatment

**Figure 7:**
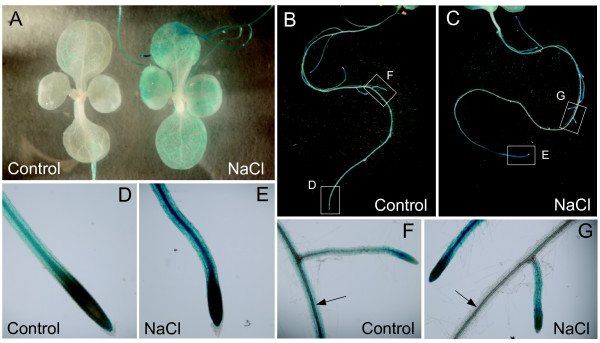
**Histochemical analysis of *POP2 *promoter activity upon NaCl treatment**. Ten-day-old plantlets of homozygous transgenic plants harbouring *pPOP2::GUS *construct grown on agar medium were transferred for 2 days on agar medium supplemented, or not (Control), with 150 mM NaCl before GUS staining. **(A) **GUS staining pattern in shoots of plantlets. **(B-C) **GUS staining pattern in roots of plantlets shown in A. **(D-E) **Focus on root apices visible in B and C. **(F-G) **Focus on areas under white boxes visible in B and C. Arrows point to primary root.

Ten-day-old homozygous transgenic plantlets harbouring *pPOP2::GUS *construct (see Methods section) were subjected to 150 mM NaCl treatment for 2 days before GUS staining. Three independent lines were investigated and showed the same GUS staining patterns but with different intensity. Under control conditions, *POP2 *was mainly expressed in roots since no GUS staining was visible in shoots (figure 7A) whereas a strong staining was present in roots (figures 7B, D and 7F). Additionally, GUS staining was present along primary and secondary roots except in the division zone of root apex (figures 7B, D and 7F; for more details see Additional file 5). In salt-treated plants, GUS staining was visible in expanded cotyledons and leaves (figure 7A). This induction of *POP2 *may be a response to Na^+ ^accumulation in shoots and suggests that the enhanced *POP2 *expression measured by qRT-PCR (figure 2C) was partly due to induction of the gene in shoots. GUS staining pattern of NaCl-treated roots seemed to be more complex. GUS staining was indeed sharply reinforced in the terminal part of primary and secondary roots, especially in the central cylinder (figures 7C, E and 7G), while coloration disappeared in the central part of primary root (figures 7C and 7G).

## Discussion

### GABA levels control upon NaCl treatment involves transcriptional and biochemical events

The accumulation of GABA in response to NaCl exposure is a common feature of plants as reported in alfalfa [39], tomato [40] and tobacco cells [41]. Until today, the molecular and biochemical events at the origin of this accumulation were misunderstood. Here, we showed in *A. thaliana *that GABA level changes under salt conditions were accompanied with variations of *in vitro *enzymes activities and transcription of GABA metabolism genes. Overall, GABA metabolism was found to be activated by NaCl treatment since almost all genes of this metabolism and both *in vitro *GAD and GABA-T activities were up-regulated (figure 2). These results basically implicate GABA metabolism in salt responses of *A. thaliana *and also suggest that metabolic flux through this metabolism is of importance under stressful conditions. However, the determination of *in vitro *GAD and GABA-T activities failed to explain GABA level changes during the first days of NaCl treatment. Indeed, within the 2 first days, GAD activity was not found to be significantly enhanced in salt-treated plantlets, even was decreased after 24 h of NaCl exposure, while in the same time GABA level and GABA-T activity were found to be significantly increased. In this context, attention should be paid to the catalytic properties of plants GADs that are known to be tightly regulated at the post-translational level by Ca^2+^/Calmodulin complex [28,29,42]. Such post-translational regulation of GAD activity should be responsible for the rapid accumulation of GABA observed in response to cold and wounding [17,43] and is likely to explain the discrepancy observed between *in vitro *GAD activity and GABA level evolutions given that NaCl treatments are known to trigger rapid elevation of cytosolic Ca^2+ ^concentration [44]. Thus, GABA accumulation in the first time of NaCl exposure would mainly result from an activation of GAD activity by Ca^2+ ^release in the cytosol; when stressful conditions are extended, GABA level control would implicate transcriptional regulation of GABA metabolism genes.

Transcriptional profiling of GABA metabolism genes demonstrated that almost all genes involved in GABA metabolism whose expression was detectable were up-regulated in response to NaCl (figure 2G). Among the three *GAD *genes whose expressions were detected, two paralogs were shown to be significantly up-regulated during NaCl treatment (*GAD2 *and *GAD4*; figure 2G). *GAD2 *expression has been shown to be ubiquitous in plant organs and to vary depending on nitrogen nutrition of plant suggesting involvement of this isoform in nitrogen metabolism [37]. Therefore, the increase of *GAD2 *expression at high NaCl concentration might be due to the necessity to adjust nitrogen metabolism under stressful conditions rather than to a specific response to NaCl. Unlike to *GAD2*, the putative *GAD4 *isoform seemed to be NaCl-specific since we showed that its expression increased in a dose-dependent manner (figure 2G). This isoform appears to be not only NaCl-responsive but is also involved in a variety of abiotic stresses since *GAD4 *was also shown to be induced in *A. thaliana *in response to hypoxia [35], cold treatment [45] and drought stress [46]. In addition, *GAD4 *was found to be overexpressed in the ABA-deficient *nc3-2 *mutant in comparison to WT under drought stress indicating that ABA may be involved in the control of its expression [46]. Analysis of *GAD4 *expression pattern under stressful conditions may bring precious information on functions of the gene. In spite of the enhancement of two *GAD *expressions, GAD activity was shown to decrease after 24 h of treatment with 150 mM NaCl. These results could be explained by (i) a time-delay between *GAD *transcripts production and their translation, (ii) the decrease of *GAD1 *expression observed upon NaCl treatment (figure 2G). The two genes involved in GABA catabolism (i.e. *POP2 *and *SSADH*) were also found to be up-regulated at moderate and high NaCl concentrations (figure 2G). These data are consistent with a high importance of GABA catabolism upon NaCl treatment and also mean that GABA-T and SSADH steps would be coordinated, probably to prevent accumulation of the reactive succinic semialdehyde (SSA) since both enzymes are located into the mitochondrion in *A. thaliana *[26,34]. We found that *POP2 *was the most highly expressed gene involved in GABA metabolism after 24 h of treatment with 150 mM NaCl (figure 2G) and was induced both in shoots and some areas of roots upon NaCl (figure 7). Taking into account that *POP2 *coding sequence is thought to be present as a single copy in *Arabidopsis *genome [25,34], its prominent expression level suggests a pivotal function of GABA-T in GABA accumulation upon NaCl treatment. In parallel, a survey of public microarray databases reveals that *POP2 *is also responsive to osmotic stress (× 4.5), senescence (× 4.05) and ABA treatment (× 2.47) [47] indicating an overall response of this step to environmental cues.

### The *pop2-1 *mutant is oversensitive to NaCl

To elucidate the contribution of the GABA-T to *Arabidopsis *NaCl responses, we performed a functional analysis of the *Arabidopsis POP2 *gene. The first step of number of gene functional analysis is to check phenotype of the corresponding loss-of-function mutant. Hence, we used the *pop2-1 *mutant which was initially isolated and characterized for its quasi-sterility [24]. Recently, *pop2-1 *mutant has been reported to be resistant to *E*-2-hexenal [30] and to accumulate a lesser amount of alanine in roots under hypoxia [35]. Here, we demonstrated that root growth of *pop2-1 *mutant was oversensitive to ionic stress since both NaCl and LiCl induced severe phenotype in mutant whereas mannitol did not (figure 4). This oversensitivity was also monitored at the plant biomass level at a later developmental stage (figure 5B). It is noteworthy that *POP2*-overexpessing plants neither showed improved salt tolerance (Additional file 6), even fed with 10 mM GABA (Additional file 7), nor were found to exhibit special vegetative and reproductive phenotype (Additional files 6 and 8).

We can ask whether high GABA levels that occur in *pop2-1 *mutant under control and even more under NaCl conditions (figure 6A) could not be toxic. Indeed, some data suggest that GABA overproduction is deleterious for plant development as shown in tobacco plants overexpressing a truncated GAD that lacks auto-inhibitory calmodulin binding site (GADΔC plants) [48]. However, since the GABA accumulation observed in these transgenic plants was also associated with a huge decrease of glutamate pool, authors did not conclude to a possible deleterious effect of GABA [48]. Arguments in favour of a non-toxic effect of high GABA levels are found in the literature as reported by Mirabella et al. [30] who associated high GABA levels to resistance to *E*-2-hexenal in *A. thaliana *either in wild-type plants fed with exogenous GABA or in the constitutively GABA accumulating *pop2*/*her1 *mutants. Moreover, Ludewig et al. [31] also ruled out the hypothesis of a higher oxidative stress induced by high GABA level in *pop2 *mutants since GABA accumulation was not shown to be associated with high reactive oxygen intermediates content. These findings are consistent with our observations indicating non-deleterious effects of 10 mM exogenous GABA on WT plantlets both under control and NaCl conditions (data not shown).

Previous works showed that GABA seemed to have a tight link with Na^+ ^transport as shown in mammals where GABA is cotransported with Na^+ ^and Cl^- ^[49] and in *A. thaliana *which was found to overaccumulate Na^+ ^when fed with GABA [50]. These observations led us to hypothesize that *pop2-1 *oversensitivity to NaCl would be due to Na^+ ^and/or Cl^- ^overaccumulation. However, determination of Na^+ ^and Cl^- ^in plantlets subjected to NaCl treatment did not reveal any difference between *pop2-1 *and its WT (figures 5C and 5D), thus invalidating our hypothesis. In contrast, K^+ ^was found to be present in a significantly lesser amount in mutant compared with its WT after NaCl treatment (figure 5E). This decrease may explain *pop2-1 *oversensitive phenotype in response to NaCl since a similar, but more severe, behaviour has been observed in the mutant of the *Salt Overly Sensitive 1 *locus [4]. Nevertheless, the *pop2-1 *mutant was found to be able to grow on low K^+ ^medium (Additional file 3), while *sos1 *mutant did not, and the K^+^/Na^+ ^ratio in mutant was not shown to be different from that of WT (data not shown). All these data suggest that K^+ ^homeostasis in the mutant would not be so far disturbed. Finally, Armengaud and coworkers [51] showed that under low K^+^, *Arabidopsis *roots accumulated carbohydrates while organic acids content decreased. Such metabolites evolutions are not similar to those observed in *pop2-1 *mutant (figure 6) indicating that the mutant did not experiment K^+ ^deficiency under NaCl treatment.

### GABA-T links N and C metabolisms in roots upon NaCl treatment

Recently, a significant effort has been done to elucidate metabolic functions of GABA in higher plants [15]. Several evidences make sense with the idea that GABA metabolism in *A. thaliana *is highly active in roots, readily more than in shoots. First, we found that GABA was about 10-fold more abundant in roots than in shoots in WT plants under control conditions (figure 2B). This observation corroborates findings of Miyashita and Good [35] in hydroponically grown *Arabidopsis *plants. Besides, in accordance with previous results obtained by qRT-PCR [34], *POP2 *was shown to be mostly expressed in roots under control conditions (figure 7) suggesting that GABA degradation occurred at a high rate in this organ. Furthermore, GAD1, a root-specific GAD responsible for the maintenance of GABA level in roots, has been characterized in *Arabidopsis *[36] whereas no shoot-specific isoform is to date identified. Overall, these data lead us to assert that GABA metabolism would be of prime importance in roots.

The great inhibition of primary root growth triggered by NaCl treatment in *pop2-1 *mutant was accompanied with substantial changes in roots metabolite profiles of mutant in comparison to WT, and these changes appeared to be more important in roots than in shoots as revealed by PCA (figure 6B). These results argue in favour of a prominent metabolic function of GABA-T in roots under NaCl conditions. This assertion is also consistent with the *POP2 *expression pattern which was found to be tightly reconfigured in NaCl-treated roots (figure 7). Metabolic changes in *pop2-1 *mutant roots included accumulation of amino acids and decrease in carbohydrates (figure 6C) strongly suggesting a function for GABA-T, and *in extenso *for GABA metabolism, in the central C/N metabolism. Several studies have reported the fluctuations of GABA content [18,52,53] or the induction of GABA-TP encoding gene [54] along day or senescence also indicating a function for GABA metabolism in C/N control. Furthermore, Fait et al. [15] found positive correlation of *GAD *and *SSADH *genes with several genes involved in central metabolism using the entire NASC0271 matrix. Overall, these findings give support to the fact that GABA plays a critical role in linking N and C metabolisms. Intriguingly, TCA cycle intermediates were not found to be present in a significant lesser amount in roots of *pop2-1 *(figure 6C and Additional file 4 for absolute values) although GABA metabolism has been thought to play an anaplerotic function [14,17]. Such a function is supported by recent studies that investigated plants impaired in TCA cycle enzymes. In these experiments, GABA was often present at a differential level in enzyme impaired-plants indicating that GABA metabolism was regulated depending on TCA cycle activity and/or integrity. These results concerned plants compromised in enzymes involved in steps both up [21,55,56] and down [57] to succinate production. The sharp decrease in carbohydrates content in *pop2-1 *roots upon NaCl treatment may be due to the necessity to compensate GABA metabolism impairment by providing increased amount of pyruvate to TCA cycle through glycolysis, which has been shown to be functionally associated with the mitochondrion [58]. We attempted to rescue *pop2-1 *phenotype by supplementing NaCl enriched medium with either 2% sucrose or with the combination of 10 mM alanine and 10 mM succinate, but attempts failed (data not shown) suggesting that metabolic impairment would not be the unique reason of *pop2-1 *phenotype.

In this context, we cannot exclude that a signaling effect of GABA would mediate *pop2-1 *oversensitivity. Indeed, GABA has been thought to act as a signaling factor in plants [17,59]. It has been shown to regulate nitrate uptake in *Brassica napus *[22] suggesting a function in regulation of nitrogen metabolism. Furthermore, GABA was found to down-regulate several *14-3-3 *genes in a Ca^2+^-, ethylene- and ABA-dependent manner [23]. Given that 14-3-3 are regulatory proteins involved in development, metabolism and stress responses [60] and that GABA reached high levels upon NaCl treatment in *pop2-1 *mutant (figure 6A), we can assume that these proteins would mediate metabolic changes recorded in the mutant.

## Conclusions

Investigation of GABA metabolism regulation upon NaCl treatment at the metabolite, enzymatic activity and gene transcription levels brought new insights into its involvement in salt responses in *A.thaliana*. We provided evidences that GABA-T step was a key point of regulation of GABA metabolism under NaCl treatment. Functional analysis of the GABA-T encoding gene *POP2 *revealed that it constituted a determinant of salt tolerance since the loss-of-function *pop2-1 *mutant was shown to be oversensitive to ionic stress in spite of higher GABA levels in its tissues suggesting that GABA itself was not associated with tolerance. Promoter-gene strategy and metabolite profiling data demonstrated that GABA-T was of prime importance in roots upon NaCl, especially linking N and C metabolisms.

## Methods

### Plant material and growth conditions

Seeds of *Arabidopsis thaliana *L*er *accession (wild-type, WT) and *pop2-1 *mutant (L*er *background) [24] were provided by the Nottingham *Arabidopsis *Stock Centre. Seeds were surface-sterilized and sown on 1% (w/v) sterile agar medium pH 5.7 (5 mM MES, Tris) in 12 cm square plates. The nutrient medium, based on Hoagland salts (half-strength for macronutrients), contained 2.5 mM Ca(NO_3_)_2_, 2.5 mM KNO_3_, 1 mM MgSO_4_, 0.5 mM KH_2_PO_4_, 53.9 μM FeNa-EDTA, 32.3 μM H_3_BO_3_, 10.6 μM MnSO_4_, 1.2 μM ZnSO_4_, 1.2 μM Na_2_MoO_4_, 0.8 μM CuSO_4 _and 0.5 μM Co(NO_3_)_2_. After 2-3 days at 4°C in the dark to synchronize germination, plates were moved to a growth room at 22°C having a 12 h-light period (light intensity of 100 μmol.m^-2^.s^-1^) and 60% relative humidity. Plates were kept vertically and their tops were not wrapped to allow transpiration.

Treatments were carried out by supplementing nutrient agar medium with compound before autoclaving. Four-day-old seedlings (Boyes' stage 1.0) [61] or 10-day-old plantlets (Boyes' stage 1.02) were individually transferred to new agar plates. Age of plants was defined with respect to the end of cold treatment. Transfer was always performed 6 h after light onset in order to take into account circadian rhythms of plants.

### Primary root measurements

To determine the effect of salts and osmoticum on primary root growth, mutant and wild-type seedlings were germinated on agar plates. Four days later, seedlings were transferred to salt- or osmoticum-supplemented plates and primary root apex was marked. Plates were photographed 6 days after transfer and root elongation was measured using the ImageJ software http://rsbweb.nih.gov/ij/. For each treatment, roots from 16 to 18 plants distributed over three plates were measured.

### Ion content

Ion content was determined in 14-day-old plantlets grown on agar medium that had been transferred for 4 days on agar plates supplemented with 150 mM NaCl. Entire plantlets were harvested, abundantly rinsed in 4 successive baths of ultra-pure water, quickly blotted and snap-frozen in liquid nitrogen. Samples (15 plants) were freeze-dried and milled with 4 mm steel balls at 20/s frequency for 2 min. Ions were extracted from ~5 mg of dry material in 1 ml of 60°C ultra-pure water for 60 min under agitation. Following centrifugation at 20000 *g *for 10 min, supernatants were recovered and diluted for ions analysis. Na^+ ^and K^+ ^were quantified using a Sherwood model 410 flame photometer (Sherwood Scientific, Cambridge, UK). Cl^- ^was quantified by ionic chromatography on a Dionex DX120 (Dionex corporation, Sunnyvale, CA) with an AS9HC column and ion Pac AG9 HC precolumn. Ions were eluted by Na_2_CO_3 _buffer and detected by conductimetry.

### RNA isolation and quantitative RT-PCR analysis

Total RNA was isolated from 30 mg of fresh material using the SV Total RNA Isolation Kit (Promega Corporation, Madison, WI) following the manufacturer's protocol. Quantity, quality and integrity of each RNA sample was assessed spectrophotometrically with a Nanodrop ND 1000 and by visualising RNA on ethidium bromide stained agarose gel. Samples were treated by DNaseI using the TURBO DNA-*free *kit (Applied Biosystems Inc, Foster City, CA). Reverse transcription (RT) was performed in 10 μl with an oligodT primer on 200 ng total RNA using the Taqman^® ^Reverse Transcription Reagents kit (Applied Biosystems Inc, Foster City, CA) according to the manufacturer's recommendations.

Primers were designed with Primer3Plus online software http://www.bioinformatics.nl/cgi-bin/primer3plus/primer3plus.cgi with qPCR settings. Care was taken to ensure that primer pairs match all known splice variants. Reverse electronic-PCR http://www.ncbi.nlm.nih.gov/sutils/e-pcr/reverse.cgi was then performed for each selected primer pair to check for single bands and correct size amplification on *Arabidopsis *transcriptome and to determinate size amplification on *Arabidopsis *genome. Primer pairs matching these requirements were tested on dilution series of either cDNA (1/10, 1/40, 1/160, 1/640, 1/2560) or genomic DNA (5 ng, 0.5 ng, 0.05 ng, 0.005 ng) to generate a standard curve and evaluate their PCR efficiency, which ranged from 92% to 109% (list of primer pairs is visible in Additional file 1).

Quantitative PCR reactions were performed on 384-wells plate in 10 μl, comprising 2 μl of 40-fold diluted RT reaction, 300 nM final concentration of each primer and *Power*SYBR Green PCR Master Mix (Applied Biosystems Inc, Foster City, CA). Plates were filled with PCR reagents using the epMotion 5070 automated pipetting system (Eppendorf, Hamburg, Germany). Corresponding RT minus controls were concurrently performed with each primer pair. PCR conditions were as follows: 95°C, 10 min; 40 × [95°C, 15 s; 60°C, 1 min] and a final dissociation step to discriminate non-specific amplifications. All reactions were performed in triplicate with the 7900HT Fast Real-Time PCR System (Applied Biosystems Inc, Foster City, CA) and data were analyzed with the SDS 2.3 software provided by the manufacturer. *PP2AA3 *gene (*At1g13320*) [62] was used as internal standard. Relative gene expression was calculated using the 2^-ΔCt ^equation, where ΔCt = Ct_*target gene *_- Ct_*PP2AA3*_.

### GABA-TP and GAD activities

Ten- to fourteen-day-old plantlets were harvested, weighed and snap-frozen in liquid nitrogen. Samples were stored at -80°C until processing. Proteins extraction and enzyme assays were performed according to Miyashita and Good [35] with some modifications.

For GABA-TP assay, protein extraction was performed in an extraction buffer containing 100 mM Tris-HCl (pH 8.0), 5 mM EDTA, 1.5 mM dithiothreitol (DTT), 1% (v/v) protease inhibitor cocktail (Sigma, #P9599) and 10% (v/v) glycerol. Four volumes of extraction buffer (v/w) and 1% (w/w) polyvinylpyrrolidone (PVPP) were added to samples before homogenization with a 4 mm steel ball at 30/s frequency for 2 min. Samples were then centrifuged at 20000 *g *for 20 min at 4°C. Supernatant was used for the enzyme assay and protein quantification. Enzyme assay was performed with 15 μl of protein extract (~30 μg of protein) in a reaction buffer containing 50 mM Tris-HCl (pH 8.0), 1.5 mM DTT, 0.75 mM EDTA, 0.1 mM pyridoxal-5-phosphate (PLP), 10% (v/v) glycerol, 16 mM GABA and 4 mM of pyruvate in a final volume of 150 μl. Control assays were concurrently performed by replacing native enzyme extract by boiled enzyme extract in the assay. After incubation at 30°C for 60 min, samples were incubated at 97°C for 7 min to stop the reaction. GABA-TP activity was evaluated by quantifying the amount of L-alanine produced by enzymatic assay using alanine dehydrogenase (AlaDH). AlaDH assay was performed with 40 μl of the GABA-T assay in an assay mix containing 50 mM sodium carbonate buffer (pH 10.0), 1 mM β-NAD^+ ^and 0.02 units of *Bacillus subtilis *AlaDH (Sigma, #A7653) in a final volume of 200 μl. The increase of OD_340 nm _was recorded using 96-well microplate reader. For each sample, a duplicate determination of alanine was done and OD_340 nm _recorded for the corresponding control was subtracted. The amount of L-alanine was calculated according to external calibration curve of L-alanine.

For GAD assay, protein extraction was performed as described above in an extraction buffer containing 100 mM Tris-HCl (pH 7.5), 1 mM EDTA, 1% (v/v) protease inhibitor cocktail (Sigma, #P9599) and 10% (v/v) glycerol. Enzyme assay was performed with 15 μl of protein extract (~30 μg of protein) in a reaction buffer containing 150 mM potassium phosphate (pH 5.8), 0.1 mM PLP and 20 mM L-glutamate in a final volume of 150 μl. Control assays were conducted as previously described. After incubation at 30°C for 60 min, samples were heated at 97°C for 7 min to stop the reaction. GAD activity was evaluated by quantifying the amount of GABA produced by enzymatic assay using GABase (Sigma). GABase assay was performed with 20 μl of the GAD assay in an assay mix containing 75 mM potassium pyrophosphate (pH 8.6), 3.3 mM 2-mercaptoethanol, 1.25 mM β-NADP^+^, 5 mM 2-ketoglutarate and 0.02 units of *Pseudomonas fluorescens *GABase (Sigma, #G7509) in a final volume of 200 μl. The increase of OD_340 nm _was recorded using 96-well microplate reader. For each sample a duplicate determination of GABA was done and OD_340 nm _recorded for the corresponding control was subtracted. The amount of GABA was calculated according to external calibration curve of GABA.

Protein concentrations were determined by the Bradford method [63] with bovine serum albumin as standard.

### Metabolites determination

Plant samples were harvested and immediately snap-frozen in liquid nitrogen. Samples were freeze-dried and then homogenized with 4 mm steel balls for 1 min at 25/s frequency. Dry plant powder was suspended in 400 μl of methanol containing 200 μM DL-3-aminobutyric acid (BABA) and 400 μM ribitol as internal standards and agitated at 1500 rpm for 15 min. Subsequently, 200 μl of chloroform were added and samples were agitated for five additional minutes. Finally, 400 μl of ultra-pure water were added, samples were then vigorously vortexed and centrifuged at 13 000 *g *for 5 min. Two aliquots of upper phase per samples were transferred to clean microtubes and dried *in vacuo*.

For amino acids analysis, dry residues were suspended in ultra-pure water and 10 μl of the resulting extract were sampled for amino acids derivatization according to the AccQTag Ultra Derivitization Kit protocol (Waters Corporation, Milford, MA). Amino acids were analysed using an Acquity UPLC^® ^system (Waters Corporation, Milford, MA) by injecting 1 μl of the derivatization mix onto an Acquity UPLC^® ^BEH C18 1.7 μm 2.1 × 100 mm column heated at 55°C. Amino acids were eluted at 0.7 ml.min^-1 ^flow with a mix of 10-fold diluted AccQTag Ultra Eluent (A; Waters Corporation, Milford, MA) and acetonitrile (B) according to the following gradient: initial, 99.9% A; 0.54 min, 99.9% A; 6.50 min, 90.9% A, curve 7; 8.50 min, 78.8% A, curve 6; 8.90 min, 40.4% A, curve 6; 9.50 min, 40.4% A, curve 6; 9.60 min, 99.9% A, curve 6; 10.10 min, 99.9% A. Derivatized amino acids were detected at 260 nm using a photo diode array detector. Amount of amino acids was expressed in μmoles per g of dry weight of sample (μmoles.g^-1 ^DW) making reference to BABA signal, external calibration curve of amino acids and dry weight of samples.

For GC-MS analysis, dry residues were dissolved in 50 μl of freshly prepared methoxyamine hydrochloride solution in pyridine (20 mg/ml). Samples were agitated for 90 min at 30°C, 50 μl of *N*-methyl-*N*-(trimethylsilyl)trifluoroacetamide (MSTFA; Sigma, #394866) were then added and derivatization was conducted at 37°C for 30 min under agitation. After transfer to glass vials, samples were incubated at room temperature over-night before injection. GC-MS analysis was performed according to Roessner et al. [64]. GC-MS system consisted of a TriPlus autosampler, a Trace GC Ultra chromatograph and a Trace DSQII quadrupole mass spectrometer (Thermo Fischer Scientific Inc, Waltham, MA). Chromatograms were deconvoluted using the AMDIS software v2.65 http://chemdata.nist.gov/mass-spc/amdis/. Metabolites levels were expressed in relative units making reference to ribitol signal and dry weight of samples.

### Plasmids construction

All PCR amplifications were conducted with *PfuUltra*™ II Fusion HS DNA polymerase (Stratagene Inc, La Jolla, CA). Amplified fragments were sequenced when introduced in either pDONR221 or pMDC32. All Gateway^® ^technology-related procedures were done according to the manufacturer's instructions.

*POP2 *promoter::GUS reporter construct was generated by amplification from L*er *genomic DNA of a promoter fragment from -1636 bp up-stream to 9 bp down-stream of the start codon of *POP2 *(*At3g22200*) using the forward primer 5'-GGGG**ACAAGTTTGTACAAAAAAGCAGGCT**GAGTTCACTAAATTCTCCTGAC-3' and the reverse primer 5'-GGGG**ACCACTTTGTACAAGAAAGCTGGGT**GCGATAACGACCATTTTCTCCTAC-3' (*att*B1 and *att*B2 sites are respectively highlighted in bold). The resulting PCR fragment was cloned into pDONR221 vector by BP clonase (Invitrogen Corporation, Carlsbad, CA) reaction and subsequently transferred into pMDC162 binary vector [65] by LR clonase (Invitrogen Corporation, Carlsbad, CA) reaction. The resulting plasmid was designated *pPOP2*::*GUS*. For *POP2 *surexpression, *POP2 *ORF [GenBank:AY142571] carried by the Gateway clone G09523 from the Salk institute was transferred to pMDC32 binary vector [65] by LR clonase reaction. The resulting plasmid was designated 2×35S::*POP2*. Binary vectors were introduced in *Agrobacterium tumefaciens *strain C58 pMP90 by electroporation.

### Plant transformation and selection of homozygous transgenic lines

Transgenic plants were generated by floral dip [66] of *Arabidopsis *(L*er*). *pPOP2*::*GUS *or 2×35S::*POP2 *constructs were used to transform T_0 _generation and T_1 _seeds were harvested in bulk, sown and screened on agar plates containing 15 mg/L hygromycin B. Hygromycin B-resistant plants were planted on soil, and the T_2 _seeds were harvested from individual T_1 _plants. The number of integrated T-DNA copies was indicated by segregation of the hygromycin B-resistance phenotype in T_2 _progeny. Transgenic lines showing a Hyg^R^:Hyg^S ^ratio of 3:1 were considered to be single-locus for the T-DNA insertion. T_3 _homozygous transgenic lines were used for the analysis of the promoter reporter gene histochemical localisation and physiological characterization.

### Histochemical staining of GUS activity

For histochemical staining of GUS activity, plant material was washed twice in a solution containing 50 mM potassium phosphate buffer pH 7.0, 0.5 mM ferrocyanide, 0.5 mM ferricyanide and 0.1% Triton X-100. Plant material was then *vacuum *infiltrated for 10 min with the same solution supplemented with 0.5 mg/ml X-Gluc substrate before incubation at 37°C. Care was taken to manipulate control- and treated-plants at the same time. After appropriate staining, chlorophyll was removed by washing leaves three times in 75% ethanol.

### Statistical analysis

Non-parametric Mann-Whitney *U*-test, Duncan multi-range test and principal component analysis (PCA) were performed using Statistica software v7.1 (StatSoft, Tulsa, OK, USA). Zero values from signal below detection limit were replaced by an arbitrary very small value (0.0001) for subsequent PCA. This concerned only HO-Proline and Trehalose levels under control conditions in shoots of both WT and *pop2-1*.

## Authors' contributions

HR, AEA and CD conceived the study and designed experiments. HR, VA and MA performed the experiments. HR, AEA and CD carried out analysis and interpretation of experimental data including statistical analyses. HR, AEA, DR, AB and CD participated to the writing of the manuscript. All authors read and approved the final manuscript.

## Supplementary Material

Additional file 1 **List of verified primer pairs used for qRT-PCR analysis**. Sequence accessions used for primers design are indicated. Click here for file

Additional file 2 **Response of *pop2-1 *mutant to various kinds of toxic cations**. Phenotype of 10-day-old plants treated for 6 days with, or without (Control), 1 mM spermidine or 100 μg/ml kanamycin. Scale bar = 1 cm. Experiment was performed three times with same results. Click here for file

Additional file 3 **Growth of *pop2-1 *mutant under low K^+ ^conditions**. Phenotype of 10-day-old plants grown on agar media containing 500, 50 or 5 μM potassium. Potassium was deleted from nutrient solution by replacing KNO_3 _and KH_2_PO_4 _with NH_4_NO_3 _and NH_4_H_2_PO_4 _respectively, potassium concentration was set by addition of KCl. Scale bar = 1 cm. Experiment was performed twice with same results. Click here for file

Additional file 4 **UPLC- and GC-MS-based metabolite profiling dataset**. Absolute values of metabolites levels are given in this excel sheet. Click here for file

Additional file 5 ***pPOP2::GUS *expression pattern in primary root apex**. Histochemical analysis of *POP2 *promoter activity in primary root apex under control conditions. Click here for file

Additional file 6 **Molecular and physiological characterization of *POP2*-overexpressing lines**. (A) *POP2 *expression in 11-day-old plantlets WT and the three 2 × 35S::*POP2 *lines. Stars indicate a significant difference with WT according to non-parametric Mann-Whitney *U*-test (*P *< 0.05). (B) GABA content in 14 day-old plantlets of WT and two *POP2 *overexpressing lines treated, or not (Control), with 150 mM NaCl for 4 days. Stars indicate a significant difference with WT according to non-parametric Mann-Whitney *U*-test (*P *< 0.05). (C) Root growth of WT and the three 2 × 35S::*POP2 *lines on agar medium supplemented, or not (Control), with 150 mM NaCl (NaCl) or 300 mM mannitol (Mannitol). Different letter indicate a significant difference according to Duncan multi-range test (*P *< 0.01). Root growth was determined as reported for figure 3. (D) Phenotype of 60-day-old plants of WT, *pop2-1 *mutant and the three 2 × 35S::*POP2 *lines alimented since their 14-day-old stage with standard nutrient solution supplemented, or not (Control), with 50 mM NaCl. Scale bar = 5 cm. Click here for file

Additional file 7 **Primary root growth response of POP2-overexpressing lines to NaCl and GABA**. Primary root growth of *POP2*-overexpressing lines on agar plates supplemented, or not (Control), with 150 mM NaCl (NaCl), or 150 mM NaCl + 10 mM GABA (NaCl+GABA). Experimental procedures are the same as reported in figure 4. Different letters indicate a significant difference with WT according to Duncan multi-range test (*P *< 0.01). Click here for file

Additional file 8 **Phenotype of siliques of *POP2*-overexpressing plants**. Phenotype of siliques of 60-day-old plants alimented since their 14-day-old stage with standard nutrient solution supplemented, or not (Control), with 50 mM NaCl. Scale bar = 0.5 cm. Click here for file
