# Supplementary material for: The Arabidopsis pop2-1 mutant reveals the involvement of GABA transaminase in salt stress tolerance
Source: BMC Plant Biol. 2010 Feb 1;10:20. doi: 10.1186/1471-2229-10-20 (PMC2825238; doi:10.1186/1471-2229-10-20)
Supplement: Additional file 1 — List of verified primer pairs used for qRT-PCR analysis. Sequence accessions used for primers design are indicated. [file 1471-2229-10-20-S1.PDF]

Target gene	Sequence accession	Forward	Reverse
GAD1	NM_121739	GGCAAGTGGAGGATTCATTG	TTTCTCCAGATCACCCAACC
GAD2	NM_001124084	GAGATGCTACGTCGTTTTGG	TGATGACAACACGCAGAACC
GAD3	NM_126261	ACGCCAGAGGAGACGCAAAG	CGCCGTTCTTGTCAGTCTTAGTG
GAD4	NM_126262	GCTGATTCGTCTTGGATTCG	AAACGCCACTAACGGAACAC
GAD5	NM_112657	CAGGATTGCACATCTTGCTG	CCACAAGGCGTTTCCAATAC
POP2	NM_113117	TGTCTCCACCGCTCATTATCTCAC	TTCCGTTGCCTTCAATGCTTTCC
SSADH	NM_106592	TTCTCCTCCACTCGTATCCTTC	CCTCCGAATCCTTATTATGTTCC
P5CS1	NM_129539.2	TTGTGATCCCAAGAGGAAGC	CGCTTTGCCATATCCGTATC
PP2AA3	NM_101203	GAGTTTGGTCCTGAATGGGCAATG	ACTGGAGCGAGAAGCGATACTG
