# Supplementary figures and images for: The Arabidopsis pop2-1 mutant reveals the involvement of GABA transaminase in salt stress tolerance
Source: BMC Plant Biol. 2010 Feb 1;10:20. doi: 10.1186/1471-2229-10-20 (PMC2825238; doi:10.1186/1471-2229-10-20)

WT

*pop2-1*

Control

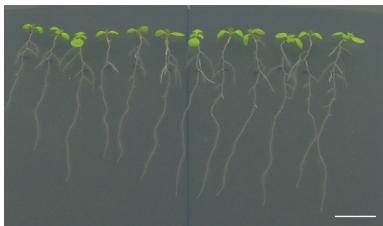

1 mM  
Spermidine

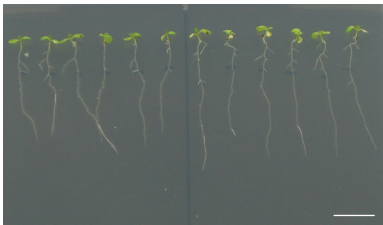

100  $\mu$ g/ml  
Kanamycin

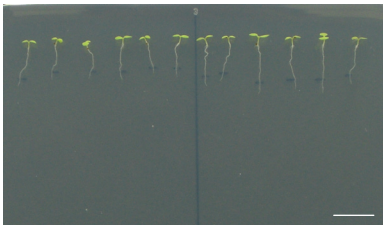

Supplement: Additional file 2 — Response of pop2-1 mutant to various kinds of toxic cations. Phenotype of 10-day-old plants treated for 6 days with, or without (Control), 1 mM spermidine or 100 μg/ml kanamycin. Scale bar = 1 cm. Experiment was performed three times with same results. [file 1471-2229-10-20-S2.PDF]

WT

*pop2-1*

500  $\mu\text{M}$   $\text{K}^+$

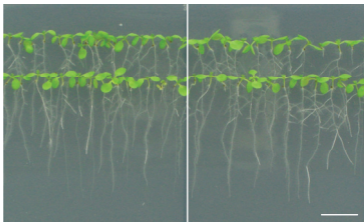

50  $\mu\text{M}$   $\text{K}^+$

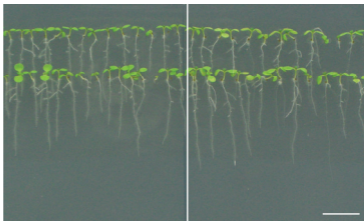

5  $\mu\text{M}$   $\text{K}^+$

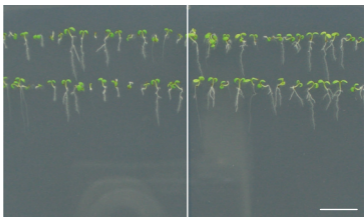

Supplement: Additional file 3 — Growth of pop2-1 mutant under low K+ conditions. Phenotype of 10-day-old plants grown on agar media containing 500, 50 or 5 μM potassium. Potassium was deleted from nutrient solution by replacing KNO3 and KH2PO4 with NH4NO3 and NH4H2PO4 respectively, potassium concentration was set by addition of KCl. Scale bar = 1 cm. Experiment was performed twice with same results. [file 1471-2229-10-20-S3.PDF]

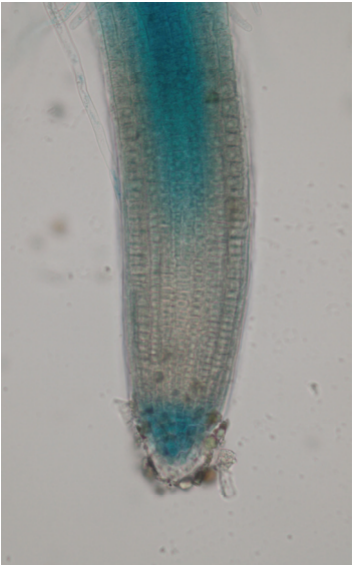

Supplement: Additional file 5 — pPOP2::GUS expression pattern in primary root apex. Histochemical analysis of POP2 promoter activity in primary root apex under control conditions. [file 1471-2229-10-20-S5.PDF]

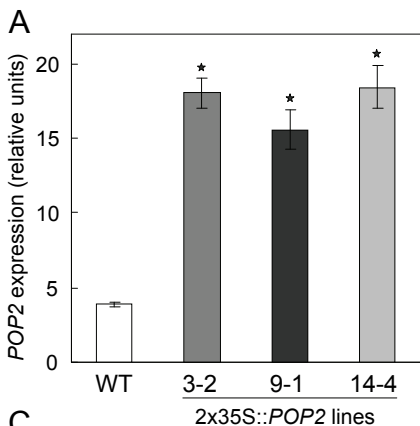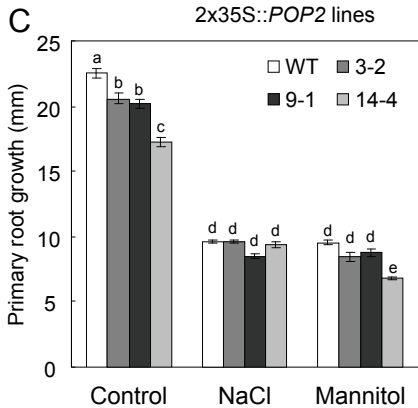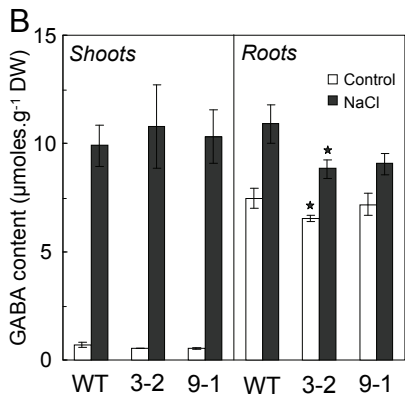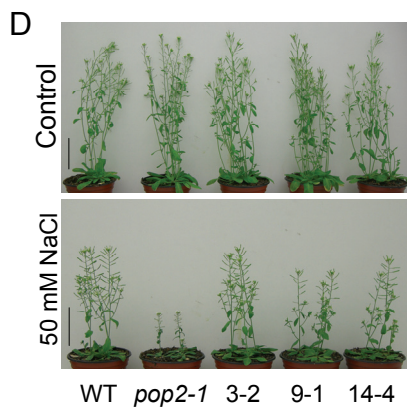

Supplement: Additional file 6 — Molecular and physiological characterization of POP2-overexpressing lines. (A) POP2 expression in 11-day-old plantlets WT and the three 2 × 35S::POP2 lines. Stars indicate a significant difference with WT according to non-parametric Mann-Whitney U-test (P < 0.05). (B) GABA content in 14 day-old plantlets of WT and two POP2 overexpressing lines treated, or not (Control), with 150 mM NaCl for 4 days. Stars indicate a significant difference with WT according to non-parametric Mann-Whitney U-test (P < 0.05). (C) Root growth of WT and the three 2 × 35S::POP2 lines on agar medium supplemented, or not (Control), with 150 mM NaCl (NaCl) or 300 mM mannitol (Mannitol). Different letter indicate a significant difference according to Duncan multi-range test (P < 0.01). Root growth was determined as reported for figure 3. (D) Phenotype of 60-day-old plants of WT, pop2-1 mutant and the three 2 × 35S::POP2 lines alimented since their 14-day-old stage with standard nutrient solution supplemented, or not (Control), with 50 mM NaCl. Scale bar = 5 cm. [file 1471-2229-10-20-S6.PDF]

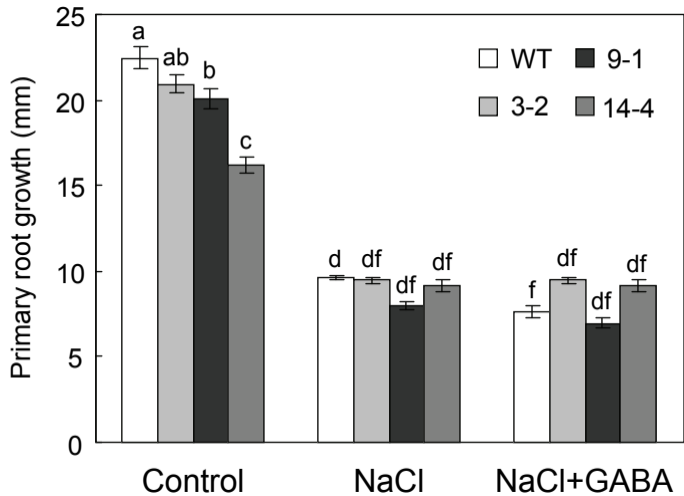

Supplement: Additional file 7 — Primary root growth response of POP2-overexpressing lines to NaCl and GABA. Primary root growth of POP2-overexpressing lines on agar plates supplemented, or not (Control), with 150 mM NaCl (NaCl), or 150 mM NaCl + 10 mM GABA (NaCl+GABA). Experimental procedures are the same as reported in figure 4. Different letters indicate a significant difference with WT according to Duncan multi-range test (P < 0.01). [file 1471-2229-10-20-S7.PDF]

WT

*pop2-1*

3-2

9-1

14-4

Control

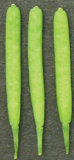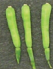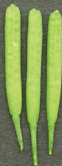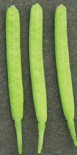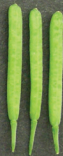

NaCl

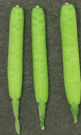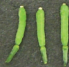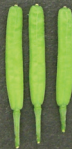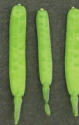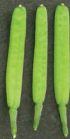

Supplement: Additional file 8 — Phenotype of siliques of POP2-overexpressing plants. Phenotype of siliques of 60-day-old plants alimented since their 14-day-old stage with standard nutrient solution supplemented, or not (Control), with 50 mM NaCl. Scale bar = 0.5 cm. [file 1471-2229-10-20-S8.PDF]
